# A Large Language Model–Powered Map of Metabolomics Research

**DOI:** 10.1021/acs.analchem.5c01672

**Published:** 2025-07-03

**Authors:** Olatomiwa O. Bifarin, Varun S. Yelluru, Aditya Simhadri, Facundo M. Fernández

**Affiliations:** † School of Chemistry and Biochemistry, 1372 Georgia Institute of Technology, Atlanta, Georgia 30332, United States; ‡ School of Computer Science, Georgia Institute of Technology, Atlanta, Georgia 30332, United States; § Petit Institute of Bioengineering and Bioscience, Georgia Institute of Technology, Atlanta, Georgia 30332, United States

## Abstract

We present a comprehensive map of the metabolomics research landscape, synthesizing insights from over 80,000 publications. Using PubMedBERT, we transformed abstracts into 768-dimensional embeddings that capture the nuanced thematic structure of the field. Dimensionality reduction with t-SNE revealed distinct clusters corresponding to key domains, such as analytical chemistry, plant biology, pharmacology, and clinical diagnostics. In addition, a neural topic modeling pipeline refined with GPT-4o mini reclassified the corpus into 20 distinct topics—ranging from “Plant Stress Response Mechanisms” and “NMR Spectroscopy Innovations” to “COVID-19 Metabolomic and Immune Responses.” Temporal analyses further highlight trends including the rise of deep learning methods post-2015 and a continued focus on biomarker discovery. Integration of metadata such as publication statistics and sample sizes provides additional context to these evolving research dynamics. An interactive web application (https://metascape.streamlit.app/) enables the dynamic exploration of these insights. Overall, this study offers a robust framework for literature synthesis that empowers researchers, clinicians, and policymakers to identify emerging research trajectories and address critical challenges in metabolomics while also sharing our perspectives on key trends shaping the field.

## Graphical abstract

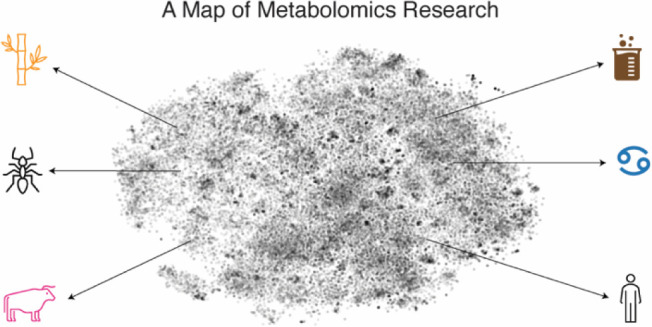

## Introduction

Metabolomics, the field that studies small molecules in biological systems, has witnessed rapid growth over the past two decades since its inception in the late 1990s.^1^ This explosion is driven by advances in analytical instrumentation, big data analysis, and increasing recognition of the profound influence of metabolites on organismal biology.^2,3^ Yet, as publication counts surge and research topics diversify—from plant metabolism^4^ to human disease biomarkers^5^—it becomes increasingly difficult for researchers to track the broadening scope of work, identify emergent themes, and discover interdisciplinary connections. Traditional literature surveys and keyword-based database queries offer only partial insights, focusing on specific research questions or narrow subfields. While these targeted approaches are useful for in-depth analyses, they are less effective at painting a panoramic picture of the research landscape. As a result, critical patterns—such as how computational methods are adopted over time, how studies cluster by organism or disease, and how novel techniques propagate across different disciplines—can remain hidden. Our endeavor to reveal such patterns within metabolomics complements broader efforts in large-scale literature analysis, such as platforms like Litmaps, by introducing a deep, domain-specific exploration powered by recent advances in language models. To capture the field’s full breadth, we curated and analyzed 80 ,656 metabolomics-related publications indexed in PubMed from 1998 through early 2024. Adapting the work of González-Márquez and colleagues on the landscape of biomedical research,^6^ in this work, we present a global framework for mapping the metabolomics literature in a two-dimensional “map.” Leveraging PubMedBERT,^7^ a domain-specific language model trained on PubMed data, we transform metabolomics-related publications into high-dimensional embeddings. We then apply dimensionality reduction methods, including t-Distributed Stochastic Neighbor Embedding (t-SNE) and Uniform Manifold Approximation and Projection (UMAP), to generate 2D layouts that capture the global structure of the embeddings. To enhance the quality of embedding interpretation, in addition to inferring the category of paper abstracts from the titles of published journals, as previously demonstrated,^6^ we conducted topic modeling using a Large Language Model (LLM)—GPT-4o mini from OpenAI—for improved distilled representation. This combination of natural language processing, LLM, and manifold learning enables a comprehensive perspective on the field, highlighting how subdisciplines and research themes align or diverge. Our approach not only confirms the prominent role of analytical chemistry and clinical studies in metabolomics but also reveals the extent to which interdisciplinary journals and diverse applications shape the research corpus. To further enrich these observations, we incorporate metadata on study designs, sample sizes, and keyword queries, allowing for fine-grained exploration of publication characteristics. The resulting visualization provides an intuitive interface for identifying high-density research clusters—such as COVID-19-related metabolomics—and for tracking how emerging areas, such as deep learning, are gaining traction over time. Additionally, to democratize access to our findings and empower broader discovery, we have developed an interactive web application (https://metascape.streamlit.app/). This platform allows users to filter and explore the map by keywords, authors, and research domains. By offering a global perspective on metabolomics, our work aims to support researchers, clinicians, and policymakers in navigating an ever-expanding literature, fostering deeper insights into our collective study of the biochemical underpinnings of life. Beyond metabolomics, this approach serves as a model for large-scale literature analysis in other fast-evolving disciplines.

## Mapping the Metabolomics Landscape: Empirical Insights and Trends

### Publication Analytics

The field of metabolomics has experienced exponential growth since its inception, with publication counts steadily increasing since 1998, to approximately 12,000 publications in 2023 in our data set (Figure 1A). The lowest rate of growth occurred between 1998 and 1999, as expected during the early stages of the field’s emergence. In contrast, the highest rate of growth was observed between 2020 and 2021, likely influenced by the COVID-19 pandemic (Table S1). The mean annual growth rate across all year pairs was approximately 448 publications. Between 1998 and 2002, the field saw consistently low growth rates, reflecting its nascent stage, while the years 2011 to 2021 exhibited consistently higher growth, highlighting the maturation and broader adoption of metabolomics techniques (Table S2). An analysis of abstract lengths (Figure 1B) shows that most abstracts contain between 150 and 250 words, with a modal length of around 200 words, consistent with standard scientific publication practices and journal word limits. A long tail of shorter and longer abstracts likely reflects the varying requirements of different publication venues. Journal frequency analysis (Figure 1C) reveals that *Scientific Reports* and *Metabolites* are the leading journals, each publishing over 2,000 papers on metabolomics. Other notable journals include *PLOS ONE*, *Analytical Chemistry*, and *International Journal of Molecular Sciences*. Additionally, a word cloud generated from the abstracts (Figure 1D) highlights key terms such as “mass spectrometry,” “identified,” “amino acid,” and “associated,” reflecting the field’s focus on metabolite identification, biochemical pathways, and quantitative techniques. The frequent use of terms such as “treatment” and “effect” underscores the clinical and applied dimensions of metabolomics, with many studies investigating the effects of interventions or treatments on metabolic profiles. Less obvious keywords like “N” and “P” indicating the number of samples and *p*-value, respectively, are also observed in the word cloud.

**Figure 1:**
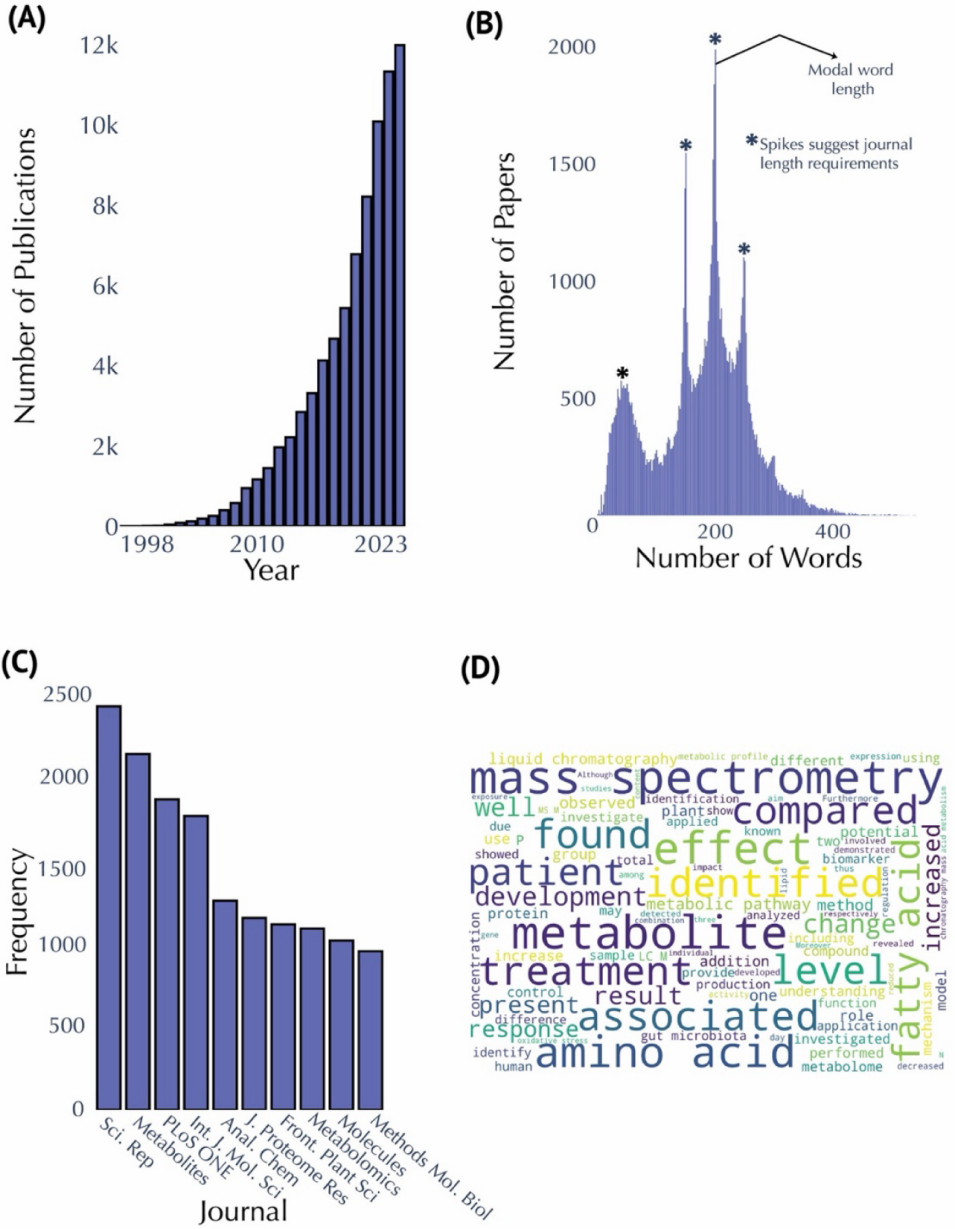
Publication and abstract analysis in metabolomics research. (A) Annual publication counts from 1998 to 2023. (B) Distribution of abstract lengths, highlighting a modal range of 150–250 words. Spikes suggested journal word limits. (C) Top ten publishing journals in metabolomics research, with *Scientific Reports* and *Metabolites* leading the field. There are 14,674 publications in the top 10 journals plotted here, which is 18.19% of the total publication. (D) Word cloud generated from abstracts; larger sized text indicates increased frequency of occurrence.

### Global View of Metabolomics Corpus

We used a domain-specific language model, PubMedBERT^7^—trained on PubMed abstracts and full-text articles—to generate 768-dimensional embedding vectors for each publication abstract. To visualize these high-dimensional embeddings, we applied two dimensionality reduction techniques: Uniform Manifold Approximation and Projection (UMAP) and t-Distributed Stochastic Neighbor Embedding (t-SNE). Both methods reduced the 768-dimensional vectors to two dimensions, providing a global perspective on how publications cluster by research domain (Figures 2A and S1). To evaluate each technique’s performance, we compared k-Nearest Neighbors (k-NN) accuracy and recall (Table 1). t-SNE outperformed UMAP on both metrics (k-NN accuracy: 0.56 vs 0.54; k-NN recall: 0.33 vs 0.14), indicating better preservation of the global embeddings in the local embedding structure. For additional context, we color-coded the reduced embeddings according to 18 predefined research categories inferred from the journal venues they are published (see Supplemental Methods for details). Analytical chemistry emerged as the most represented field (8,981 publications), followed by plant biology (4,648) and pharmacology (4,229), while nephrology (277) and sports science and medicine (76) had fewer publications (see complete breakdown in Table S3). The t-SNE visualization (Figure 2A) reveals distinct clusters corresponding to major research areas such as plant biology, analytical chemistry, environmental sciences, animal sciences, pharmacology, cancer biology, and immunology. Plant biology exhibits a compact cluster, suggesting a strong thematic focus (Figure 2B), whereas the broader spread of analytical chemistry indicates wide applicability across metabolomics (Figure 2C). Overlapping clusters—like toxicology and environmental sciences (Figure 2D,E)—highlight interdisciplinary connections, which are similarly evident for other domains like cancer research and immunology as well as animal science and food science. To broaden our “global view” of metabolomics publications beyond the 2D t-SNE (Figure 2A) and UMAP (Figure S1) embeddings used for domain clustering, we examined temporal patterns across the corpus. We color-coded the t-SNE embeddings by publication year (Figure S2), revealing how early work (pre-2010) clustered primarily around analytical chemistry and plant biology. We then divided the data set into eight discrete time periods (1998–2000 through 2022–early 2024) and generated separate t-SNE plots for each (Figure S3). Early clusters (1998–2009) are comparatively sparse and heavily oriented toward analytical methods, reflecting that analytical chemistry had the highest publication count before 2010, followed by plant biology (Table S4). In contrast, later periods exhibit denser, more diverse clusters, mirroring both the exponential growth of metabolomics and its expanding translational applications.

**Figure 2:**
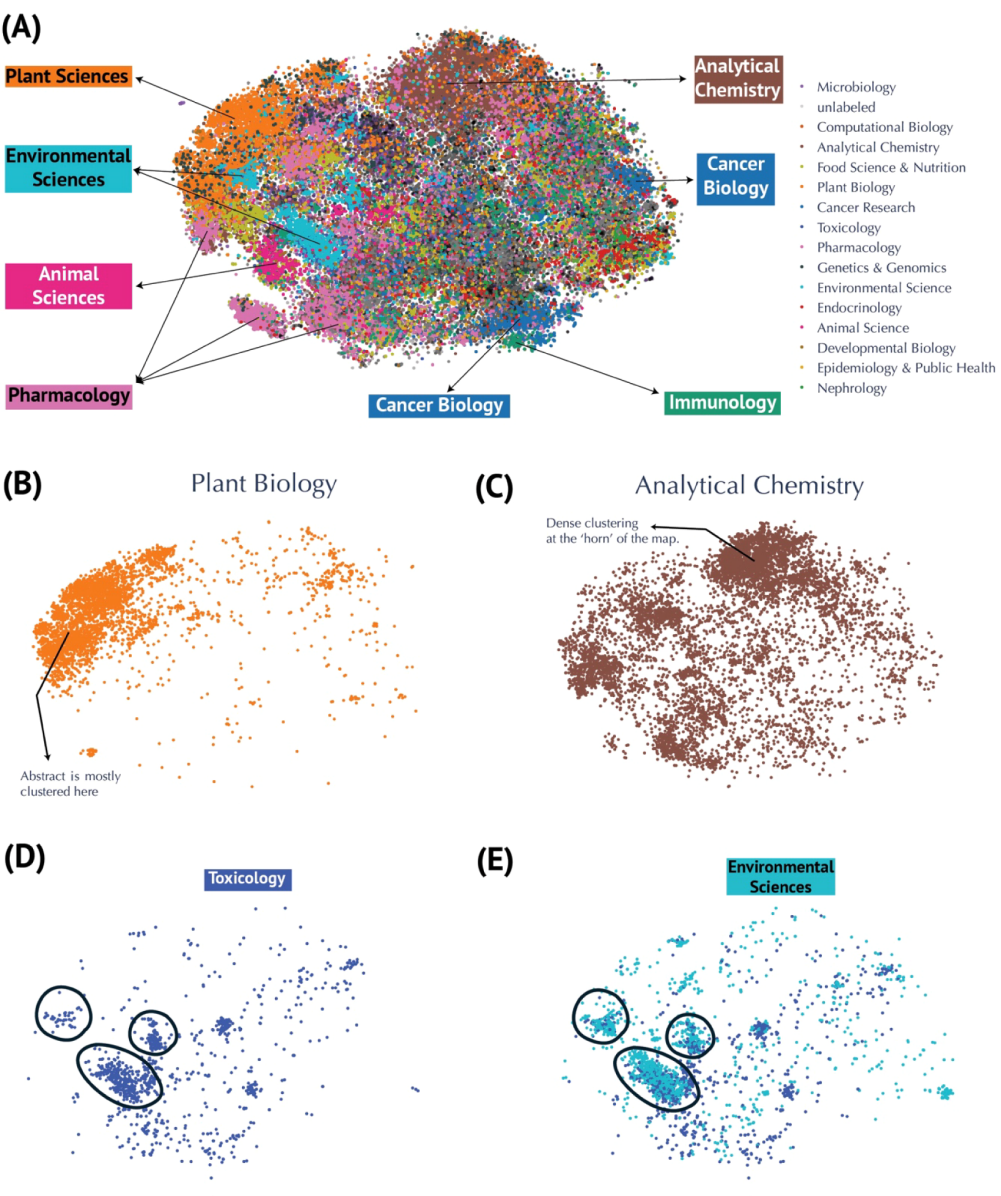
Visualization of metabolomics research fields using t-SNE embeddings. (A) Annotated t-SNE projections highlighting research domains. (B) Focused visualizations of clusters for Plant Biology and (C) Analytical Chemistry. Dense clustering is seen at the “horn” of the map. (D-,E) Examples of overlapping clusters, such as Environmental Sciences and Toxicology, illustrating connections on the map between related domains.

**Table 1:** Performance Evaluation of Dimensionality Reduction Techniques^a^

	*k*-NN Accuracy	*k*-NN Recall
*t*-SNE	0.56	0.33
umap	0.54	0.14

a Comparison of t-SNE and UMAP on preserving the structure of 768-dimensional PubMedBERT embeddings in a two-dimensional space. Metrics include k-Nearest Neighbors (k-NN) accuracy and k-NN recall.

One limitation of inferring a publication’s research domain from the title of the journal is that many articles in multidisciplinary journals go unlabeled. Indeed, the “unlabeled” cluster comprises 41,721 publications (Figure S4; Table S3) and is broadly dispersed, reflecting the heterogeneity of papers that could not be assigned to predefined categories. This wide distribution highlights how multidisciplinary or nonspecialized journals nevertheless contribute significantly to metabolomics research. To address this issue, we performed topic modeling using c-TF-IDF for topic representation and GPT-4o mini for topic fine-tuning, thereby generating thematic labels that capture each cluster’s topical scope rather than merely its research field. For instance, in the cluster representing plant biology, the c-TF-IDF computation returned keywords such as *plants*, *genes*, *compounds*, *stress*, *growth*, *species*, *leaves*, *biosynthesis*, and *production* (Figure S5). These words, together with representative abstracts, were provided to GPT-4o mini to produce more descriptive labels (see Methods for details). Overall, this approach revealed 20 distinct topics, including “Plant Stress Response Mechanisms,” “Metabolic Profiles and Dysregulation,” “Cancer Metabolism and Therapy Resistance,” “Metabolomics Data Analysis and Integration,” “Gut Microbiota and Metabolomic Interactions,” “Metabolomics in Neurodegenerative Disorders,” “Environmental Toxicology and Metabolism,” “Metabolomics in Animal Nutrition,” “Microbiota–Gut–Brain Axis Interactions,” “Kidney Disease Metabolomics and Biomarkers,” “Metabolomics in Pregnancy,” “Lung Disease and Metabolic Dynamics,” “NMR Spectroscopy Innovations in Metabolomics,” “COVID-19 Metabolomic and Immune Responses,” “Lipid Profiling Techniques,” “Host–Parasite Metabolic Interactions,” “Male Fertility and Reproductive Metabolomics,” “Ocular Metabolomics and Disease Mechanisms,” and “Salivary Metabolomics in Oral Health” (Figures S5 and 3A). In contrast to the research field embeddings, which labeled ∼40,000 publications (Table S3), the topic modeling assigned 65,676 publications to a defined topic (see complete breakdown in Table S5). To quantitatively assess the semantic quality and interpretability of the topics generated, we calculated topic coherence using the *C*_v_ metric. *C*_v_ is a widely adopted measure that has been shown to correlate strongly with human judgments of topic coherence^8^ (see Supplemental Methods for details). Our analysis revealed an average *C*_v_ score of 0.7311 across all topics, indicating a strong overall coherence for the model. A detailed breakdown of the *C*_v_ scores for each individual topic is provided in Table S6. Notably, topics such as “COVID-19 Metabolic and Immune Responses” achieved a *C*_v_ of 0.91, and “NMR Spectroscopy Innovations in Metabolomics” scored 0.86, underscoring the high semantic relatedness of keywords used, in concert with abstracts, to generate LLM labeled topics. Other coherence metrics, such as normalized pointwise mutual information, also yielded positive average scores (e.g., average *C*_npmi_ = 0.17, Table S6), providing additional support for the model’s coherence. With respect to the new thematic labels, notably, the plant biology cluster (“Plant Stress Response Mechanisms”) remains compact (Figure 3B), consistent with its presentation in the research field embeddings (Figure 2B) and contains the most assigned publications at 19,258 (Table S5). The next largest topic, “Metabolic Profiles and Dysregulation,” encompasses various forms of altered metabolism in human diseases, reflected by discriminating keywords such as *liver*, *diabetes*, *insulin*, *muscle*, *mice*, *exercise*, *obesity*, *heart*, *diet*, and *hepatic*. (Figure S5) Meanwhile, “Cancer Metabolism and Therapy Resistance” appear with scattered clusters throughout the atlas, emphasizing its interdisciplinary nature (Figure 3C). The dense analytical chemistry cluster at the “horn” of the atlas (Figure 2B) is now partly decomposed into “Metabolomics Data Analysis and Integration” (Figure 3D) and “NMR Spectroscopy Innovations” (Figure 3E), most likely indicative of method development.

**Figure 3:**
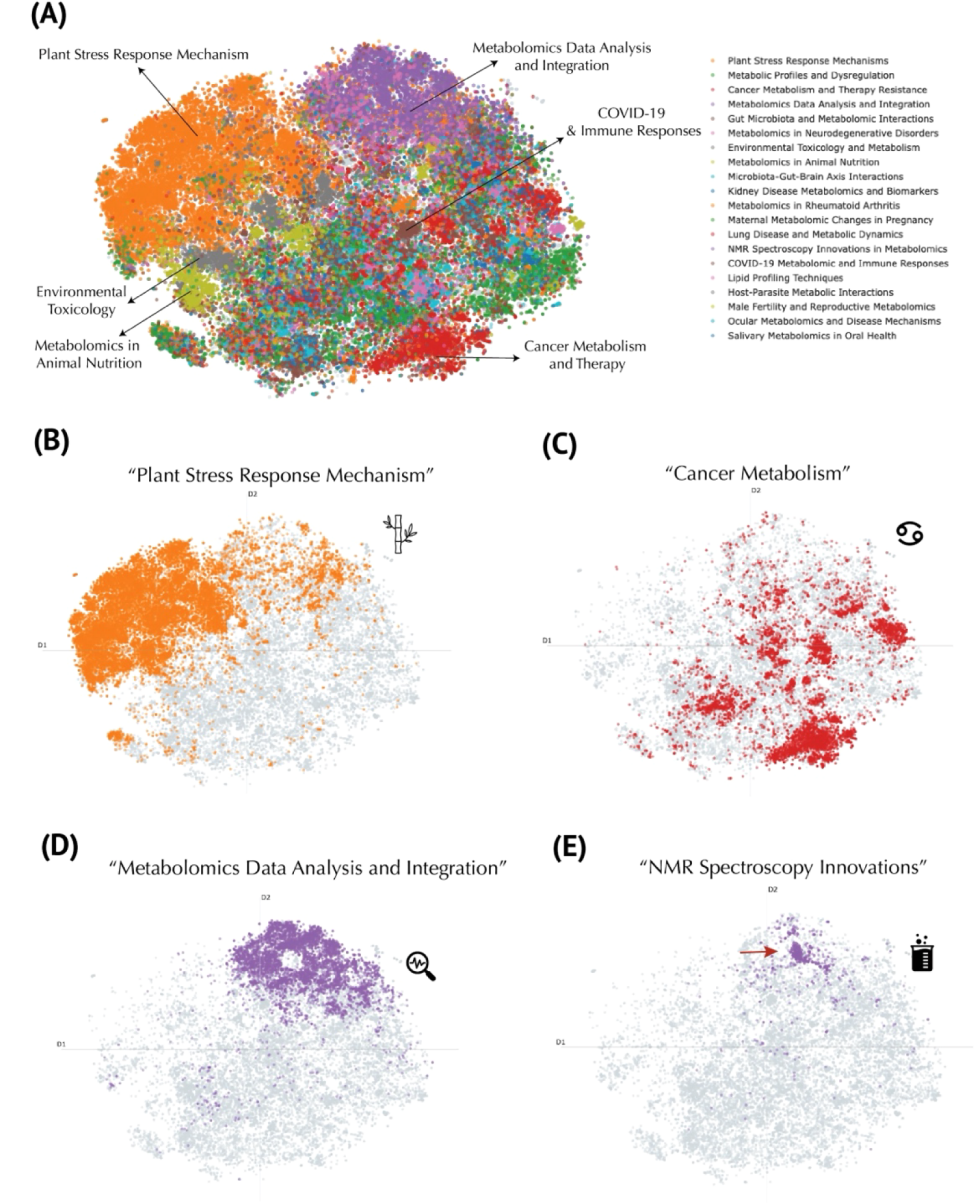
Topic modeling of the metabolomics corpus using a large language model. (A) Global t-SNE projection of publications, colored by representative topics discovered through the BERTopic pipeline. The legend (top right) lists exemplar thematic labels generated by GPT-4o mini. (B) Focus on publications labeled as the “Plant Stress Response Mechanism” (orange). (C) Cluster of “Cancer Metabolism” publications (red), illustrating the concentration of work on metabolic pathways implicated in oncogenesis and therapy resistance. (D) “Metabolomics Data Analysis and Integration” (purple), marking computationally oriented publications. (E) “NMR Spectroscopy Innovations” topic (purple), distinct from the data-integration cluster, focused on methodological advancements in nuclear magnetic resonance techniques.

### Research Trend Discovery

We leveraged our global embedding space to pinpoint emergent topics and patterns in the metabolomics literature (Figures 4). Keyword-based queries proved to be particularly revealing. For example, studies whose abstracts include the phrase “for the first time” are dispersed throughout the map, signifying a diverse range of novel contributions (Figure 4A). By contrast, research on “COVID-19 or SARS-CoV-2” clusters densely in one region (Figure 4B), highlighting the concentrated surge in metabolomics investigations related to pandemic-driven questions, with publications from 2020 onward (Figure S6). Inspection of additional metadata features offered further insights into study designs and analytical practices. *P*-values are more prevalent in disease-focused or animal science contexts (Figure 4C), suggesting that hypothesis-driven experimental designs are particularly common in these domains. Figure 4D shows reported sample sizes concentrated in clinical and animal studies with most samples under 50 (*n* = 1849; Figure 4E). Larger sample sizes—exceeding 1000—was only 51 (Figure 4E) and tends to appear in systematic meta-reviews. Figure S7a–d illustrates how searches for “biomarker discovery” and for “(pathway analysis OR metabolic pathway) AND mechanism” in the abstract occupy distinct but partially overlapping regions of the corpus. Biomarker-focused papers are widely distributed in the analytical chemistry and disease-related clusters, aligning with the mid-to-late 2000s rise in translational research, likely reflective of early publication efforts in biomarker discovery (Figure S7c,d). In contrast, mechanistic studies are particularly prominent in plant biology and pharmacology (Figure S7a) and skew slightly more recently (Figure S7b), suggesting ongoing exploration of metabolic pathways in these fields. We also contrasted classical multivariate approaches with newer machine-learning methods. While “chemometrics or multivariate analysis” is well represented across multiple decades, abstracts mentioning “deep learning or neural network” show a comparatively late surge (Figure S7e,f). A more focused query on “deep learning” alone (Figure S7g,h) reveals that although still sparse, these publications cluster in data-science–oriented regions of the map and predominantly appear after 2015. Finally, we supplemented these findings by color-coding articles according to the collaboration size, journal title length, and abstract length (Figure S8). Collaborative efforts (Figure S8a) range from small teams to large consortia, underscoring the varied scale of the research. Journal title length (Figure S8b) does not appear to correlate with any particular domain, whereas the distinct pattern seen in the abstract length (Figure S8c) likely reflects specific journal guidelines and article types. Overall, this multifaceted embedding-based analysis offers a robust framework for charting how specific research foci and methodological approaches have emerged and evolved across the metabolomics literature.

**Figure 4:**
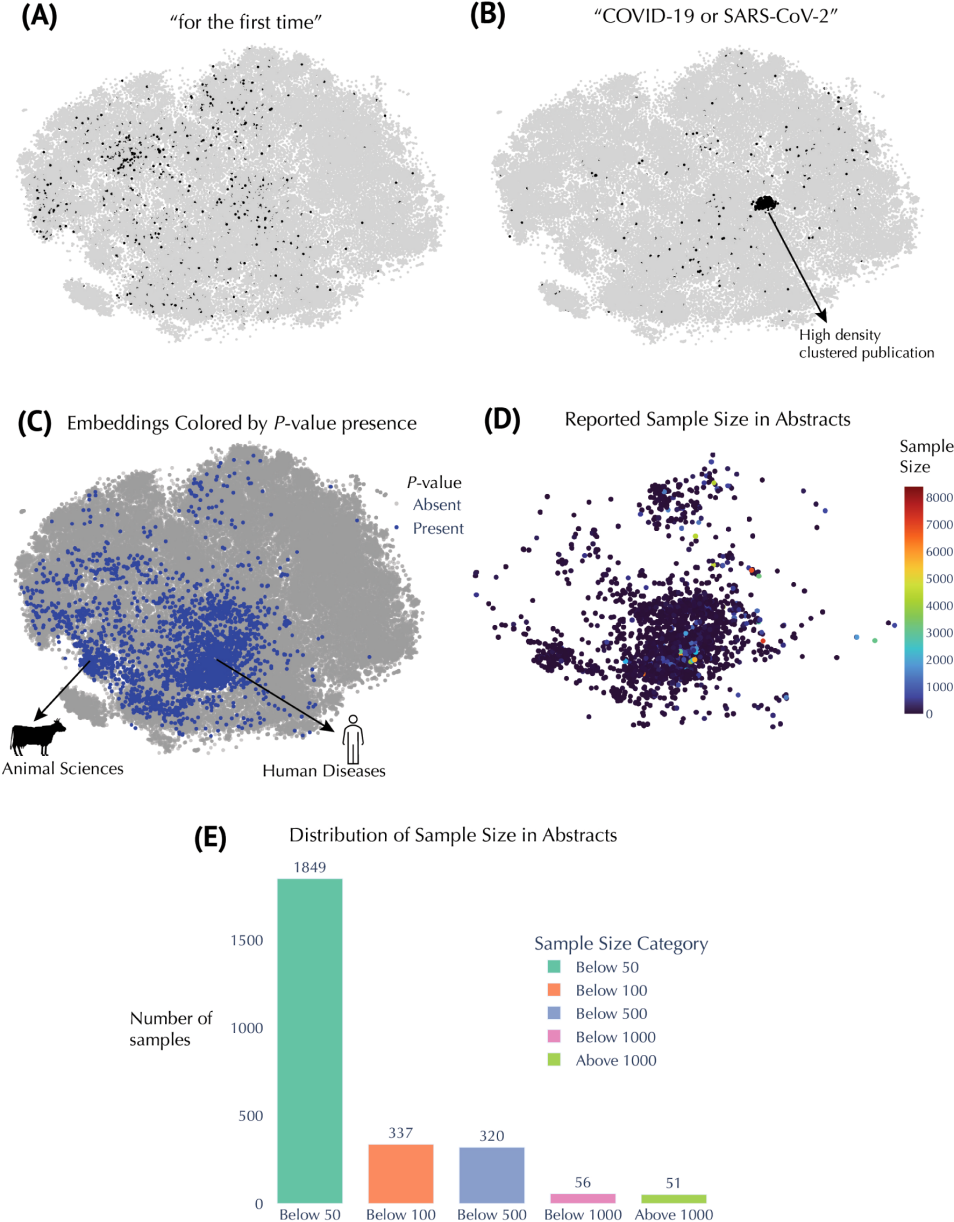
Insights from embedding metadata in metabolomics research. (A) t-SNE embeddings filtered to show abstracts containing the phrase *for the first time.* (B) t-SNE embeddings filtered for abstracts containing “COVID-19” or “SARS-CoV-2” (regex: COVID-19|SARS-CoV-2). (C) Embeddings colored by presence or absence of *P*-values, identified by the regex [pP]\s?[<=>]. For example, phrases such as “*p* < 0.05,” “*P* = 0.01,” or “*p* > 0.001” match this pattern. (D) Embeddings colored by reported sample size, extracted from expressions matching n\s?=\s?(\d+). For example, “*n*=30,” “*n* = 500,” or “*n* = 1000” would be captured. (E) Distribution of these extracted sample sizes across the relevant abstracts.

### Synthesis of Findings and Perspectives

Here, we present a comprehensive global map of metabolomics research by integrating advanced natural language processing with dimensionality reduction and topic modeling techniques. This methodology not only enables the identification of key research domains but also captures the dynamic interplay of topics that underlie the progression of the field, thereby offering critical insights that extend beyond traditional literature reviews.

One notable finding is the sustained period of growth that began in 2011. Our temporal analysis reveals that the field experienced a marked acceleration in publication output starting that year. This acceleration may be attributed to factors such as heightened interest, increased funding, technological advancements, and the exploration of broader applications.^2^ Indeed, our map (Figure S3) demonstrates that metabolomics applications expanded significantly after 2010. Overall, this growth underscores both the maturation of metabolomics and its expanding influence across diverse domains, including analytical chemistry, clinical diagnostics,^9^ and plant biology.^10^

One unsurprising finding is the prominent role of analytical chemistry within metabolomics research. Our analyses reveal that analytical chemistry remains the dominant theme, as evidenced by its extensive representation in the corpus and the widespread distribution of its associated publications across the embedding space (Figure 2C). This reflects the foundational role that sophisticated analytical methods, such as mass spectrometry^11^ and NMR spectroscopy,^12^ play in the identification and quantitation of metabolites. In contrast, the plant biology cluster exhibits a more compact and tightly focused grouping, indicating a high degree of thematic cohesion in studies centered on plant stress responses and biosynthetic pathways^10^ (Figure 2B).

A key strength of our approach is the integration of topic modeling with refined language generation. By applying a c-TF-IDF framework^13^ in conjunction with GPT-4o mini, a large language model from OpenAI, we were able to generate nuanced and informative labels for each cluster, thereby moving beyond the limitations of inferring research domains solely from journal titles^14^ (Figures 2A and 3A). This approach allowed for the reclassification of over 65,000 publications into 20 distinct topics, covering a wide range of research areas—from “Plant Stress Response Mechanisms” (Figure 3B) to “NMR Spectroscopy Innovations” (Figure 3C). While leveraging GPT-4o mini significantly enhanced the generation of informative topic labels, we acknowledge that the use of such large language models is not without limitations. These can include sensitivity to specific prompt phrasing and a theoretical risk of hallucinations, which we aimed to mitigate by providing strong contextual inputs (c-TF-IDF keywords and representative abstracts) and by utilizing structured prompting.

Furthermore, by leveraging keyword-based queries in abstracts, we were able to observe differences between two major thematic streams in metabolomics research: biomarker discovery and pathway mechanism elucidation. Biomarker-focused studies, predominantly found in analytical chemistry and clinical clusters, echo early translational efforts from the mid-2000s^15^ (Figure S7c,d). In contrast, mechanistic investigations, which are particularly prominent in plant biology and pharmacology, offer deeper insights into the biochemical pathways driving metabolic processes^16^ (Figure S7a). In addition, recent surge in studies employing deep learning for metabolomics data analysis was observed^17^ (Figure S7g,h).

Despite the impressive volume of research, a significant challenge in metabolomics is the low number of samples in many studies (Figure 4D,E), which has important consequences. For instance, while numerous investigations report promising metabolic biomarkers, there remains a scarcity of clinically validated and widely adopted biomarkers.^18^ This gap is partly attributable to issues, such as small sample sizes and insufficient validation across independent cohorts. For example, a meta-analysis on pancreatic cancer found that 87% of 655 potential biomarkers were reported in only single studies.^19^ However, it is important to note that a small number of samples, such as the modal group of less than 50 samples identified in this study, can be entirely appropriate for cell-based, animal, and methodological optimization studies; nevertheless, they also dominate many published biomarker-discovery papers, where underpowered designs remain a recognized bottleneck.

The temporal analysis conducted using our embedding framework further enhances the value of this work. By color-coding publications based on the year of publication and dividing the data set into discrete time periods, we were able to observe the evolution of research clusters over time (Figure S3). Early work in the field was predominantly concentrated on analytical methods (Figure S2b and Table S4), while more recent years show a diversification that includes translational research and clinical applications. For example, the cluster related to COVID-19 or SARS-CoV-2 metabolomics^20^ illustrates how the field can rapidly pivot in response to global health challenges, resulting in a densely populated area within the map (Figures 4B and S6). Such temporal insights afforded by this technique can be invaluable for policymakers, funding agencies, and research leaders seeking to understand the progression of scientific inquiry and identify opportunities for interdisciplinary collaboration.

Another noteworthy observation is the impact of publication venues on the dissemination of metabolomics research. Our publication analytics reveal that open-access journals such as Scientific Reports, Metabolites, and PLOS ONE collectively account for close to 50% of the top ten metabolomics publications, with the journals representing the top three (Figure 1C). This underscores the preference for open-source dissemination.

While our approach has yielded valuable insights, incorporating additional metadata such as full-text analysis, author affiliations, or funding sources could further refine our classifications and provide a more granular view of the research landscape. Looking ahead, the global mapping framework presented here holds significant promise for application in other fast-evolving subdisciplinary areas. Furthermore, the development of interactive platforms, such as our web application (https://metascape.streamlit.app/), empowers researchers, clinicians, and policymakers interested in the field of metabolomics to explore trends and identify emerging areas of interest with unprecedented ease.

The multifaceted portrait of metabolomics derived from our comprehensive analysis—reflecting its rapid expansion, the persistent centrality of analytical innovation, and burgeoning clinical and applied interests—signals a field rich in discovery yet navigating critical junctures. Observed trends, such as the increasing adoption of advanced computational methods like deep learning, alongside the enduring challenge of translating numerous biomarker candidates into robust clinical tools, underscore this pivotal moment. Our perspective is that metabolomics’ future trajectory will be significantly defined by its capacity for strategic convergence. This means fostering a more synergistic relationship between technological advancement—which includes preparing for the impending artificial intelligence explosion, where AI systems are poised to significantly contribute to scientific discovery^21^—and rigorous biological validation, as well as between high-throughput screening and deep functional investigation. Effectuating this involves not just generating more data but more effectively synthesizing existing knowledge to guide hypothesis-driven research. Furthermore, the discovery of robust disease biomarkers urgently requires large, multiinstitution consortiums and substantial sample sizes, thereby informing study design on statistical power and cohort diversity. Crucially, as biomedical discovery transitions toward an AI-enabled era, where necessary, metabolomics databases must be upgraded for seamless compatibility with AI systems. The ability to discern these macrolevel patterns and identify underexplored or overly saturated niches, as our mapping approach facilitates, is paramount for the community to collectively steer metabolomics toward realizing its profound potential in understanding and modulating complex biological systems for improved health and environmental outcomes.

## Methods

A detailed description of the methods can be found in Section S1.

### PubMed Data Retrieval

We compiled a corpus of PubMed articles by querying for the terms “metabolomics” or “metabonomics.” Data spanning 1998–early 2024 were acquired via the Entrez Programming Utilities (E-Utilities) from the National Center for Biotechnology Information (NCBI). The search looked for the selected terms in various fields of PubMed records, such as titles, abstracts, MeSH terms, and author keywords. We extracted the following key fields: PubMed ID, article title, abstract text, language, publication year, and author names. Records missing abstract text, publication year, or the last author’s name were discarded. The final curated data set consisted of 80,656 publications.

### Publication Classification and Analysis of Publication Statistics

To classify publications by research domain, we used journal titles as proxies to infer the primary focus of each article, adapting the work of González-Márquez et al.^6^ We developed a keyword mapping spanning 18 predefined categories. To standardize classification, journal titles were converted to lowercase, and publications were assigned to a category if the journal title contained any of the category’s keywords. For each abstract retrieved, publication metadata such as publication years and journal titles allowed for the assessment of growth trajectories and publication characteristics over time. Abstract length distribution was computed. We identified the top publishing journals by counting the number of metabolomics papers published in each journal. We also used word cloud visualization to highlight the most commonly occurring terms across all abstracts.

### Embeddings Generation and Dimensionality Reduction

Embeddings for publication abstracts were generated using the PubMedBERT model^7^ via the Hugging Face Transformers library. For each abstract, the final 768-dimensional embedding was computed by taking the mean of the last hidden states across all tokens. To visualize the higher-dimensional embeddings, we employed two widely used dimensionality reduction techniques: t-Distributed Stochastic Neighbor Embedding (t-SNE) and Uniform Manifold Approximation and Projection (UMAP). Both methods reduced the original 768-dimensional embeddings to two dimensions. To evaluate the effectiveness of each technique, we used two metrics: k-Nearest Neighbors (k-NN) accuracy and k-NN recall.

### Topic Modeling with a Large Language Model

We performed topic modeling on metabolomics abstracts using a pipeline that combined precomputed PubMedBERT embeddings, dimensionality reduction, and clustering with BERTopic.^13^ Clustering was conducted with HDBSCAN. Text within each cluster was vectorized using a CountVectorizer. Cluster representations were generated using BERTopic’s class-based Term Frequency-Inverse Document Frequency (c-TF-IDF) approach. To refine topic labels, we employed OpenAI’s GPT-4o-mini model through BERTopic’s integrated TextGeneration module (see prompt in Appendix B). The final outputs included topic assignments, probabilities, and refined labels.

## Supplementary Material



## Data Availability

The code used for the analysis is available at GitHub: https://github.com/obifarin/metamap. The PubMed data set and associated embeddings can be accessed on Zenodo: https://doi.org/10.5281/zenodo.15020144.^22^ The first file is a curated XML data set used in this study, covering the period from 1998 to early 2024. To ensure data quality, entries with errors have been removed. The second file contains embeddings and associated data sets within the same time frame. It includes PubMed metadata such as PMID, title, abstract, language, journal title, publication year, and authors. Additionally, we provide full 768-dimensional embeddings generated using PubMedBERT, along with UMAP and t-SNE embeddings for dimensionality reduction and visualization.

## Notes

Section S1: Supplemental Methods; Figure S1: Global visualization of metabolomics research fields using UMAP embeddings; Figure S2: Temporal distribution of metabolomics publications visualized using t-SNE embeddings; Figure S3: Time-segmented t-SNE visualizations of metabolomics research fields; Figure S4: Unlabeled Cluster of Metabolomics Publications; Figure S5: c-TF-IDF Word Score for Metabolomics Research; Figure S6: Embeddings illustrating the impact of COVID-19–related research; Figure S7: Evolution of Keyword-based Queries in the Metabolomics Corpus, Highlighting Shifts in Methodological Approaches and Conceptual Focuses Over Time; Figure S8: Embeddings colored by publication metadata features; Table S1: Derivative Analysis of Publication Trends in Metabolomics Research; Table S2: Years with Consistently High and Low Rates of Publication Change in Metabolomics Research; Table S3: Publication counts by research fields in metabolomics; Table S4: Publication counts by research fields in metabolomics from 1998 to 2009; Table S5: Publication counts by topics in metabolomics; Table S6: Per-Topic Coherence Scores for Topic Model. Appendix A: Code Showing Label Mapping for Label Inference using Journal Titles; Appendix B: Prompt for Topic Modeling (PDF)
