# Supplementary material for: A Large Language Model–Powered Map of Metabolomics Research
Source: Anal Chem. 2025 Jul 3;97(27):14088–96. doi: 10.1021/acs.analchem.5c01672 (PMC12268820; doi:10.1021/acs.analchem.5c01672)
Supplement: Supplementary file 1 [file ac5c01672_si_001.pdf]

Supporting Information

A Large Language Model–Powered Map of Metabolomics Research.

Olatomiwa O. Bifarin[1], Varun S. Yelluru[2], Aditya Simhadri[2] Facundo M. Fernández[1,3]
[1] School of Chemistry and Biochemistry, Georgia Institute of Technology, Atlanta, Georgia 30332, United States
[2] School of Computer Science, Georgia Institute of Technology, Atlanta, Georgia 30332, United States
[3] Petit Institute of Bioengineering and Bioscience, Georgia Institute of Technology, Atlanta, Georgia 30332, United States

Table of Contents

Section S1. Supplemental methods.
Figure S1. Global visualization of metabolomics research fields using UMAP embeddings.
Figure S2. Temporal distribution of metabolomics publications visualized using t-SNE embeddings.
Figure S3. Time-segmented t-SNE visualizations of metabolomics research fields.
Figure S4. Unlabeled Cluster of Metabolomics Publications.
Figure S5. c-TF-IDF Word Score for Metabolomics Research.
Figure S6. Embeddings illustrating the impact of COVID-19–related research.
Figure S7. Evolution of Keyword-based Queries in the Metabolomics Corpus, Highlighting Shifts in Methodological Approaches and Conceptual Focuses Over Time.
Figure S8. Embeddings colored by publication metadata features.
Table S1. Derivative Analysis of Publication Trends in Metabolomics Research.
Table S2. Years with Consistently High and Low Rates of Publication Change in Metabolomics Research.
Table S3. Publication counts by research fields in metabolomics.
Table S4. Publication counts by research fields in metabolomics from 1998 to 2009.
Table S5. Publication counts by topics in metabolomics.
Table S6. Per-Topic Coherence Scores for Topic Model.
Appendix A: Code Showing Label Mapping for Label Inference using Journal Titles.
Appendix B. Prompt for Topic Modelling.

Section S1: Supplemental Methods

PubMed Data Retrieval Details

To efficiently manage queries and circumvent the 10,000-record limit of the standard E-Utilities API, we employed the Entrez Direct (E-Direct) package. Two primary E-Direct commands, esearch and efetch, facilitated both the initial search and full-record downloading. Specifically, we executed: esearch -db pubmed -query "metabolomics OR metabonomics" -mindate 1998 | efetch -format xml. Key fields were extracted using a custom Python script. For simplicity, we concatenated first and last author names into a single field.

Publication Classification Details

Keywords spanned 18 predefined categories: oncology, plant biology, nephrology, endocrinology, microbiology, analytical chemistry, pharmacology, neuroscience, food science and nutrition, toxicology, environmental science, animal science, sports science and medicine, epidemiology and public health, developmental biology, aging and gerontology, immunology and vaccine research, and computational biology. Examples of keywords included "cancer," "oncogene," and "tumor" for "Oncology," and "bacteriology," "yeast," and "microbiome" for "Microbiology". Journals that did not match any category-specific keywords were labeled as “unclassified”. This resulted in 41,721 publications falling under “unclassified,” reflecting the prevalence of multidisciplinary journals. All keyword mappings are provided in **Appendix A**.

Analysis of Publication Statistics Details

Word cloud visualization used the WordCloud python library (version 1.9.3). Default stop words were removed in addition to custom stop words ‘used’ and ‘study’. To identify trends, we computed the year-over-year change rates by calculating the difference in publication counts between consecutive years. We analyzed the annual derivative of publication counts to identify periods of consistently low/high growth. The mean and standard deviation of rates of change for each starting year were calculated. The overall mean rate of change was 447.58 publications per year. Thresholds were set as: (a) High Growth: Mean rate + 1 SD, and (b) Low Growth: Mean rate - 1 SD for a given starting year. Years exceeding the high growth threshold were classified as high growth periods, and those below the low growth threshold as low growth periods. Early 2024 publications were removed from this specific analysis.

Embeddings Generation Details

The specific model used was microsoft/BiomedNLP-PubMedBERT-base-uncased-abstract-fulltext via the Hugging Face Transformers library (version 4.38.1) and implemented in PyTorch (version 2.1.0+cu121). Each abstract was preprocessed using the model’s tokenizer, with padding and truncation applied to a maximum input length of 512 tokens. A custom function passed inputs to the model on a GPU-enabled environment (NVIDIA Tesla T4, CUDA version 12.2 via Google Colab). Resulting embeddings were stored in a DataFrame as an HDF5 file.

Embeddings Dimensionality Reduction Details

The scikit-learn implementation (version 1.2.2) of t-SNE was utilized with n_components = 2 and random_state = 42. UMAP was implemented using the umap-learn library (version 0.5.5) with the

same parameters. Two metrics, k-NN Recall and k-NN Accuracy provided quantitative insights into how well reduced embeddings preserved original structure. The scikit-learn library was used for computing these metrics. k-NN accuracy assessed the proportion of correctly classified samples in the reduced space. A k-NN classifier was trained on the 2D embeddings using a train-test split (1% test set). The classifier was configured with n_neighbors = 10, algorithm = brute, and n_jobs = -1. The accuracy was then evaluated on the test set. k-NN recall measured the local neighborhood preservation between the high-dimensional and reduced-dimensional spaces. For each data point, k=10 nearest neighbors were computed in both original and 2D spaces using Euclidean distance. The common neighbors between these two sets were identified by calculating the intersection for each data point. The total number of shared neighbors was then summed across all data points. Finally, the kNN recall was determined by dividing this sum by the total number of possible neighbor comparisons (number of data points multiplied by 10), yielding a measure of neighborhood preservation. This dual-metric evaluation allowed rigorous assessment of t-SNE and UMAP performance.

Topic Modelling Details

Precomputed embeddings were integrated via a custom PrecomputedEmbeddings class. HDBSCAN (version 0.8.29) was used with min_cluster_size = 500, min_samples = 300, metric = 'euclidean', and cluster_selection_method = 'eom'. Text was vectorized using scikit-learn's (version 1.2.2) CountVectorizer, removing English stop words and capturing unigrams and bigrams appearing in at least ten abstracts. Both unigrams and bigrams were included to ensure topic specificity. BERTopic's (version 0.16.3) c-TF-IDF generated cluster representations. OpenAI’s GPT-4o-mini model (via API Client version 1.51.1) and BERTopic’s TextGeneration module refined topic labels. Representative documents (up to 100 per topic) and c-TF-IDF keywords were inputs. API efficiency was managed through built-in delays. A prompt consisting of a system prompt, an in-context example, and a main prompt was used (See Appendix B). The pipeline was executed in a high-memory, GPU-enabled environment in Google Colab. To quantitatively assess the semantic coherence of the generated topics, we calculated C_v and C_{npmi} coherence scores. This involved extracting the top ten words for each non-outlier topic identified by the BERTopic model. These sets of words were then evaluated against the entire corpus of abstracts. The abstracts were tokenized using Gensim's simple_preprocess function (version 4.3.3), and a Gensim Dictionary was created from these tokens. Finally, Gensim's CoherenceModel was employed to compute both the C_v and C_{npmi} coherence values, providing an average score for the overall model and individual scores for each topic.

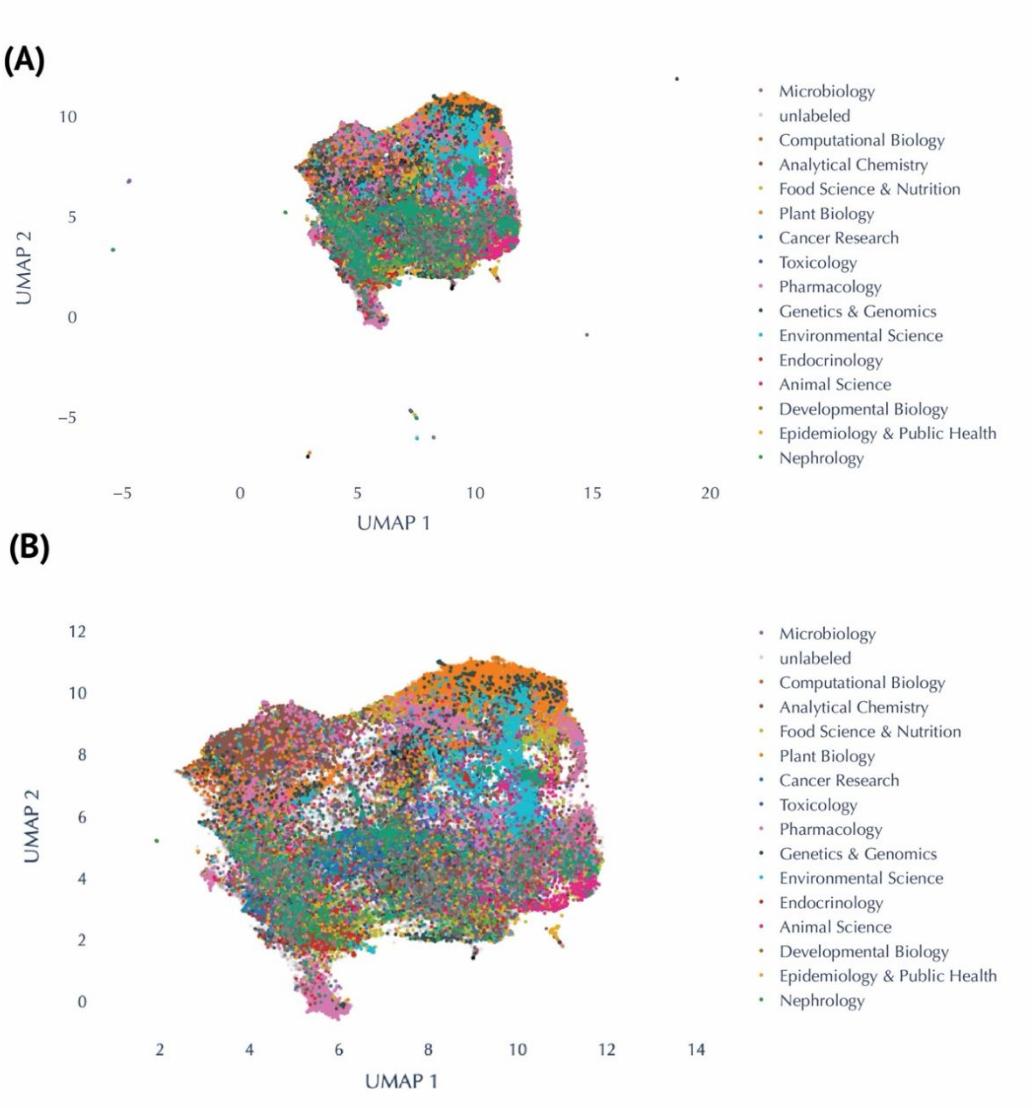

Figure S1. Global visualization of metabolomics research fields using UMAP embeddings.

(A) Two dimensional UMAP projection of 80,656 publications.

(B) A magnified view of two dimensional UMAP projection of 80,656 publications.

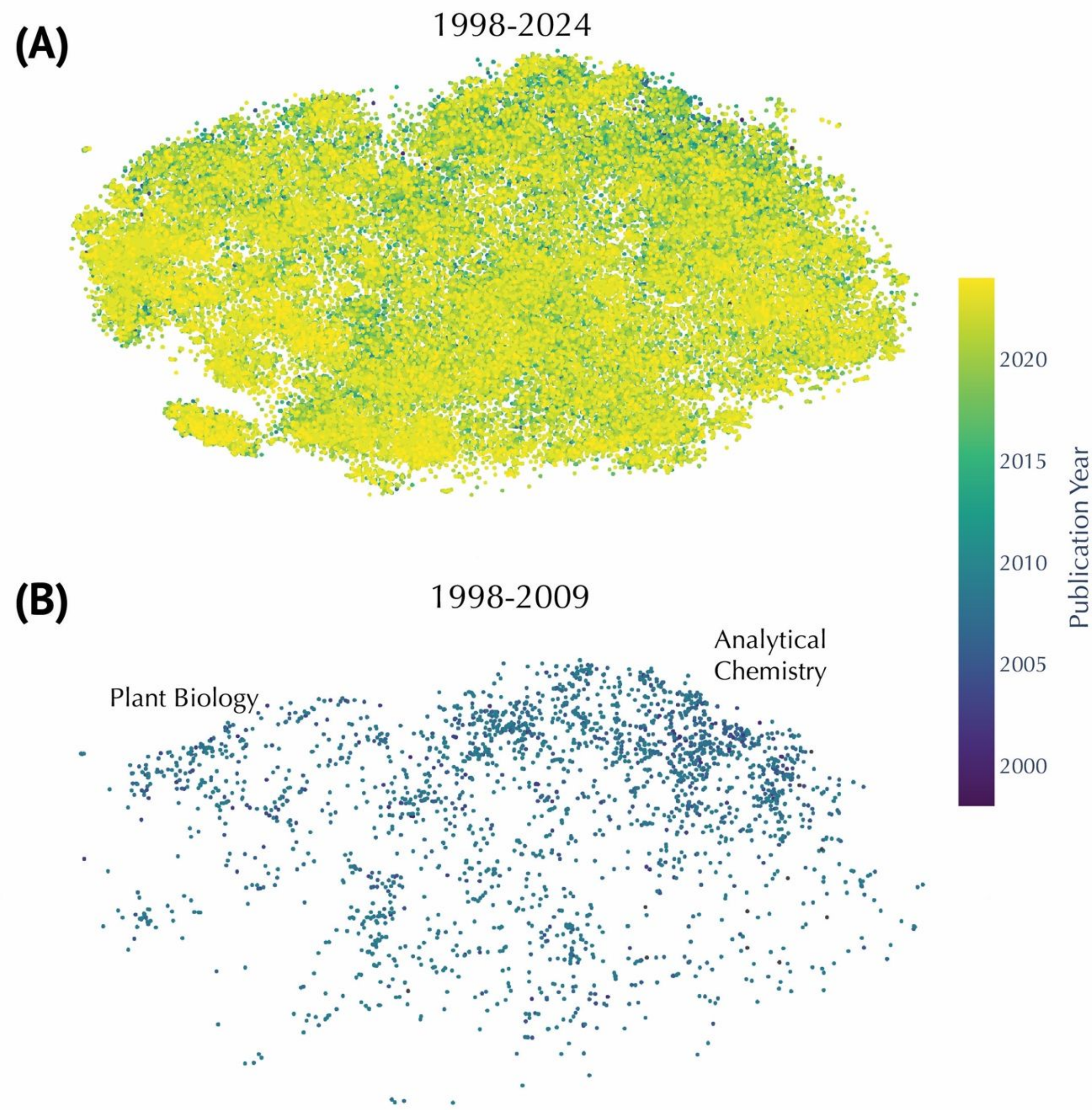

Figure S2. Temporal distribution of metabolomics publications visualized using t-SNE embeddings.
(A) Showing all publications from 1998 to early 2024.
(B) Showing all publications between 1998 and 2009. The publications are concentrated in the 'Analytical Chemistry' and 'Plant Biology' area of the map.

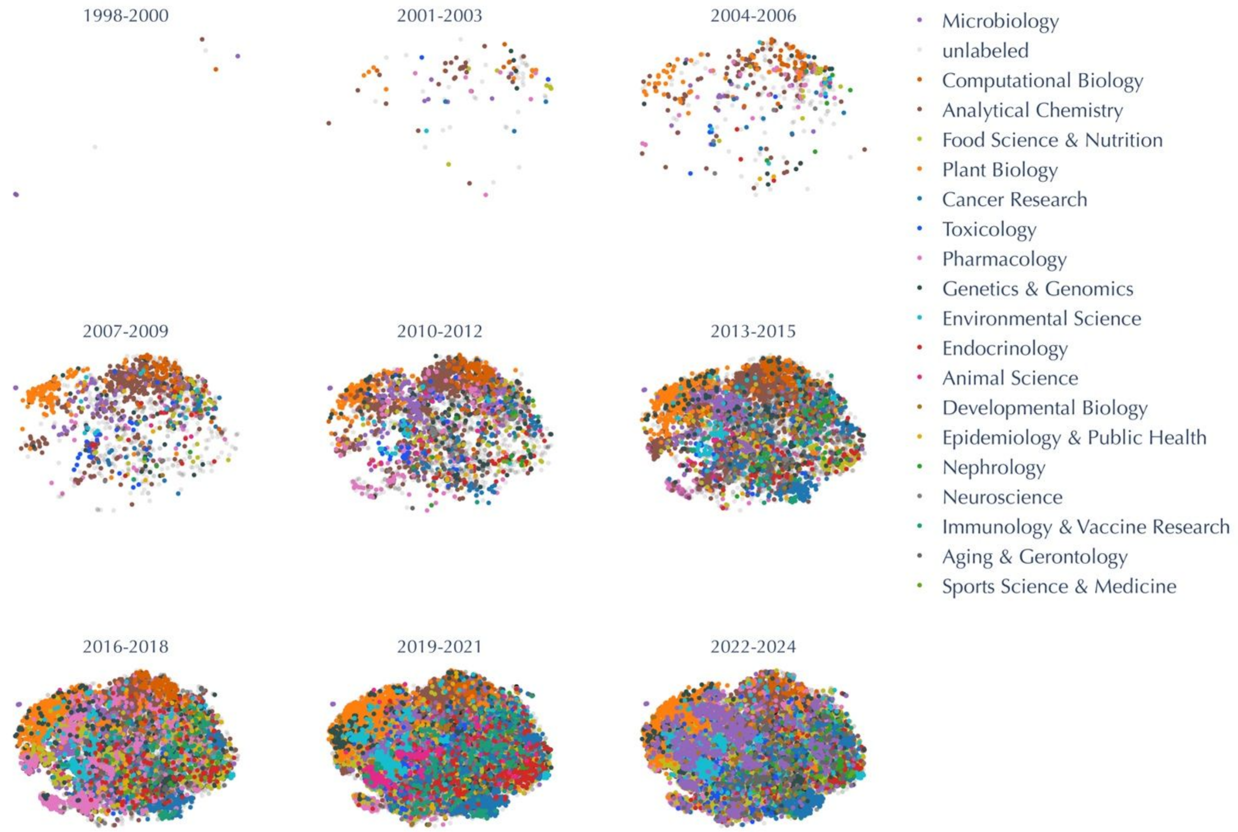

Figure S3: Time-segmented t-SNE visualizations of metabolomics research fields. Clusters represent metabolomics publications divided into eight time periods (1998–2000, 2001–2003, 2004–2006, 2007–2009, 2010–2012, 2013–2015, 2016–2018, 2019–2021, and 2022–early 2024). Each point represents a publication, color-coded by its research domain.

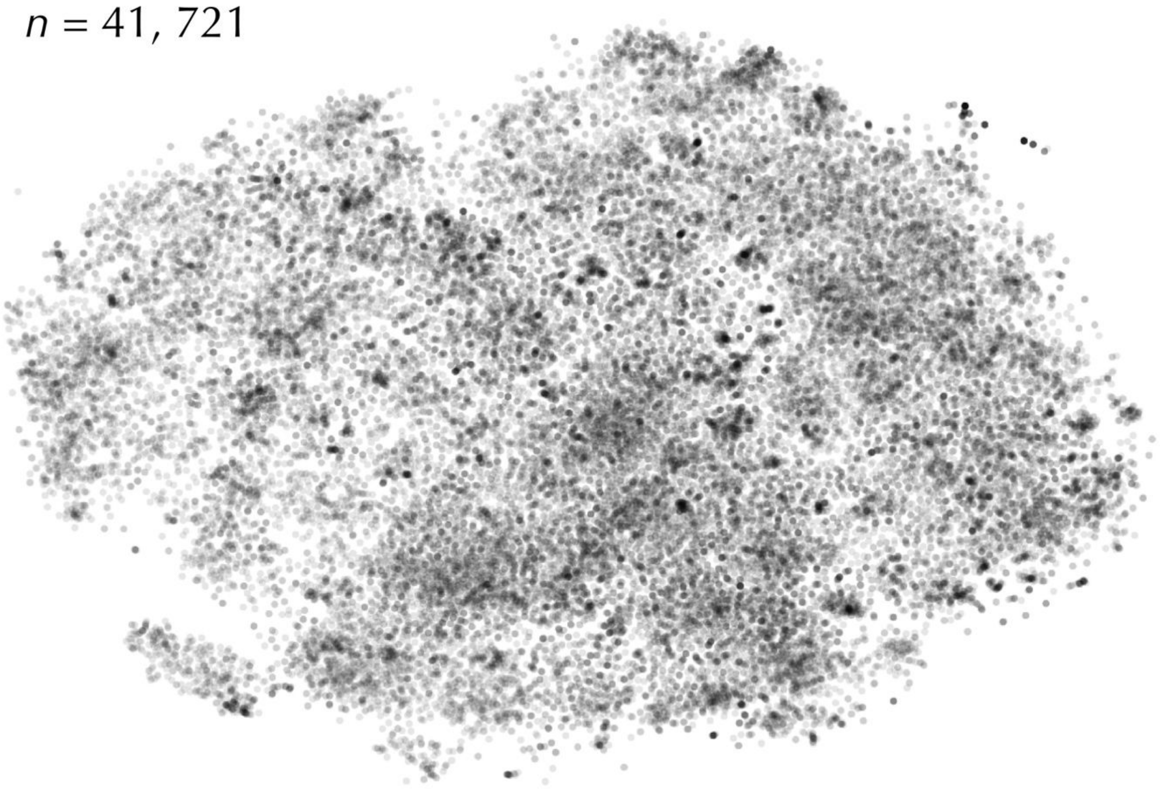

Figure S4: **Unlabeled Cluster of Metabolomics Publications.**
Scatterplot of 41,721 “unlabeled” publications in a t-SNE projection, representing articles not assigned to predefined journal-based categories.

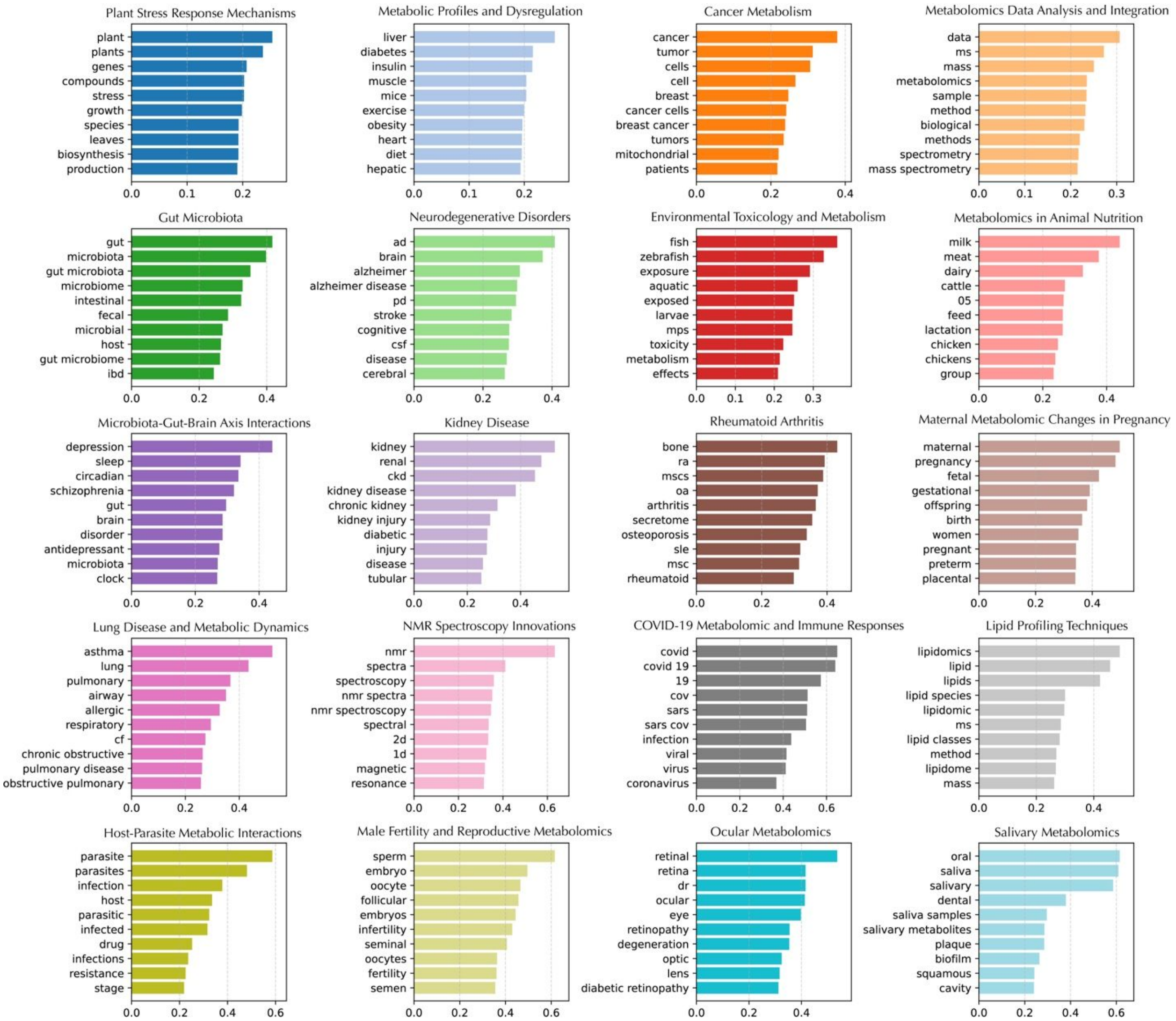

Figure S5. **c-TF-IDF Topic Word Scores for Metabolomics Corpus.** Each subplot represents a topic identified through BERTopic modeling of metabolomics literature. The x-axis shows the c-TF-IDF score, representing the importance of each word within the topic. The y-axis lists the top 10 most relevant words for each topic.

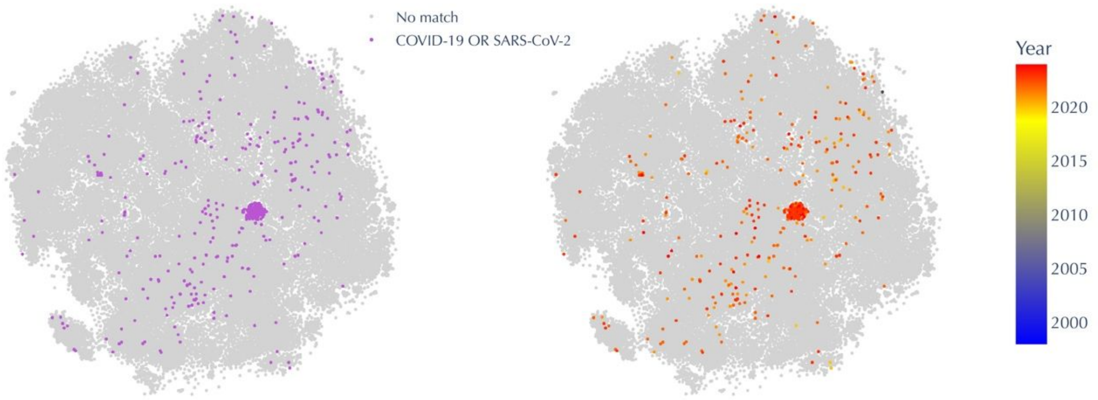

Figure S6: **Embeddings illustrating the impact of COVID-19–related research**. **Left:** Points in **purple** indicate abstracts mentioning “COVID-19 or SARS-CoV-2,” while **gray** points show no mention. **Right:** The same embedding colored by publication year, transitioning from **blue** (earlier) to **red** (more recent). The visible cluster of purple/red points emphasizes the surge of COVID-19 metabolomics studies in recent years.

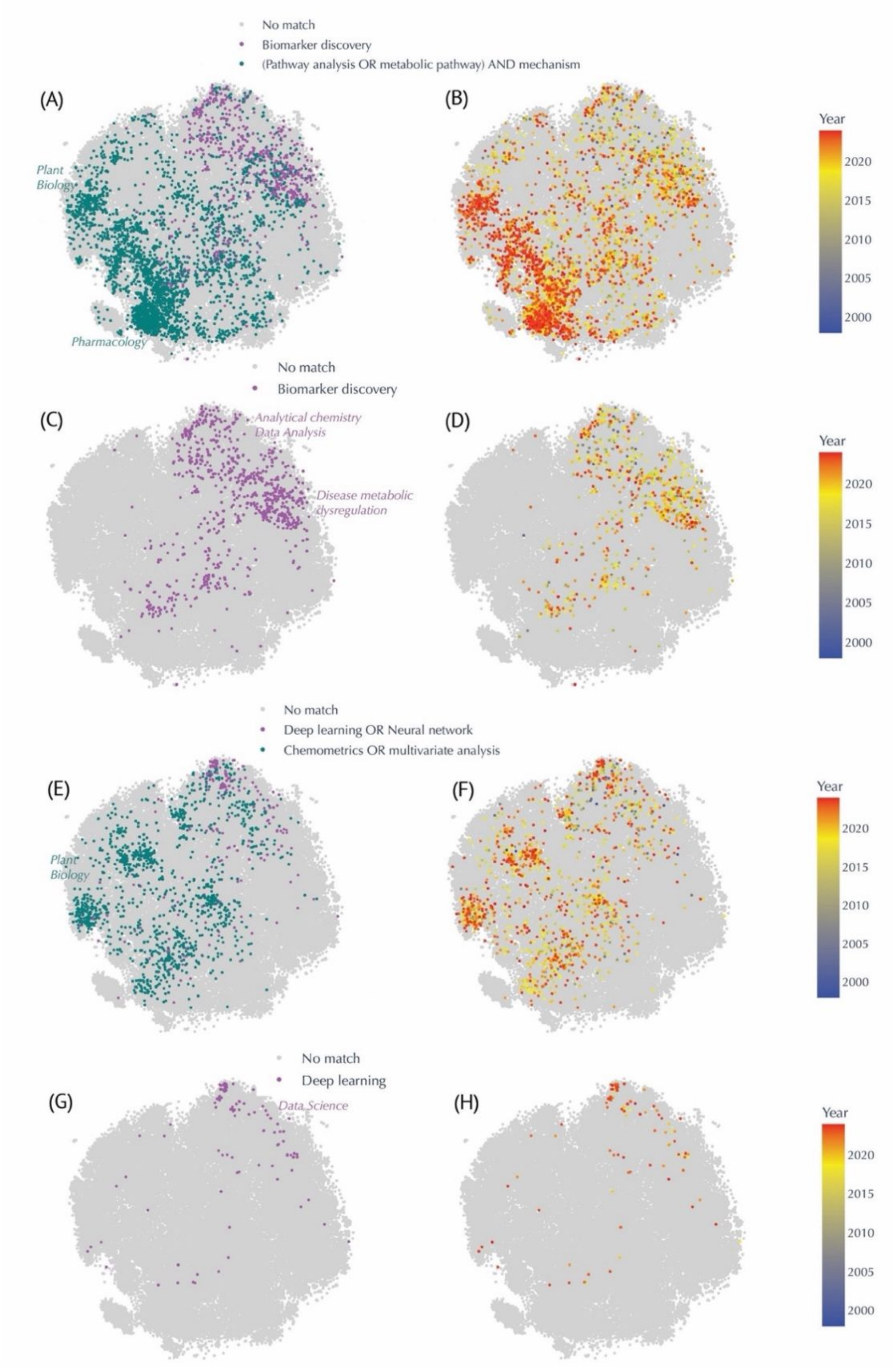

Figure S7. Evolution of Keyword-based Queries in the Metabolomics Corpus, Highlighting Shifts in Methodological Approaches and Conceptual Focuses Over Time. In each keyword panel, matched abstracts appear in color, while unmatched entries appear in light gray. The heatmap on the right panels transitions from **blue** (older publications) to **red** (more recent publications).

(A–B) Comparison between “Biomarker discovery” (**teal**) and “(Pathway analysis OR metabolic pathway) AND mechanism” (**purple**). Papers mentioning “pathway mechanisms” cluster prominently in plant biology and pharmacology regions and are more recent (B), while “biomarker discovery” studies stretch across analytical chemistry and disease-related areas from the mid-2000s onward (A).
(C–D) Focus on “Biomarker discovery” alone (purple) and its temporal distribution, revealing a surge in clinical and disease-oriented research in the late 2000s (D).
(E–F) Contrast between “Deep learning OR neural network” (**purple**) and “Chemometrics OR multivariate analysis” (**teal**). Classical multivariate methods span earlier periods, whereas deep learning publications have intensified mainly post-2015 (F).
(G–H) Spotlight on “Deep learning” (purple), showing relatively sparse coverage overall, yet relatively denser clustering in data science–focused areas in more recent years (H).

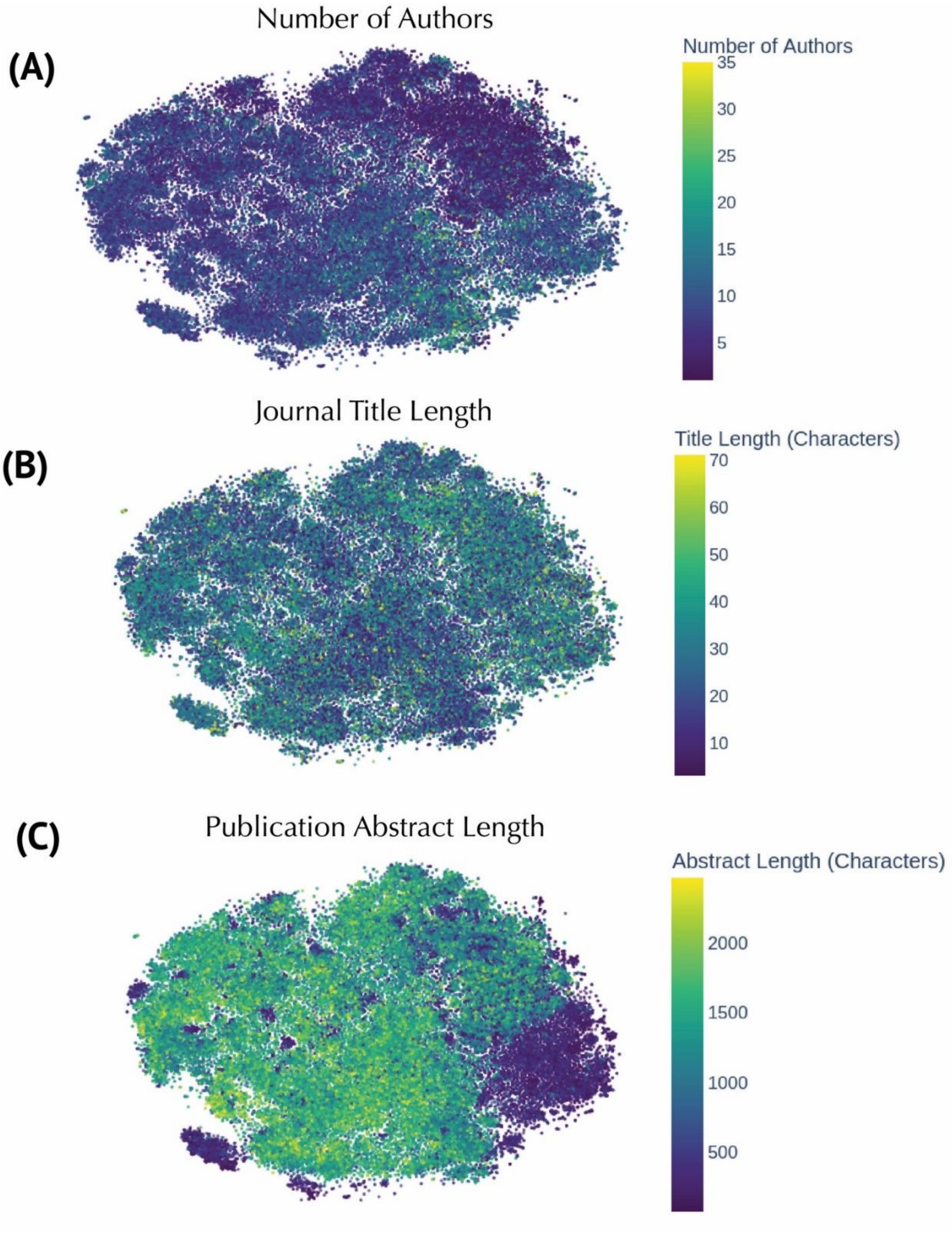

Figure S8: Embeddings colored by publication metadata features.
(A) t-SNE embeddings of metabolomics publications colored by the number of authors per publication, highlighting collaboration trends across the corpus.
(B) Embeddings colored by the length of journal titles.
(C) Embeddings colored by abstract length.

Table S1: Derivative Analysis of Publication Trends in Metabolomics Research.
Yearly derivatives were computed to identify periods of growth acceleration and deceleration. The lowest derivatives occurred during the nascent stages of the field (1998–2001), while the highest derivatives were observed during recent years (2019–2022).

Year 1	Year 2	Derivative
Lowest derivatives		
1998	1999	0
1998	2000	3.00
1998	2001	3.67
2000	2001	5.00
1999	2001	5.50
Highest derivatives		
2020	2021	1872.00
2019	2021	1653.50
2020	2022	1559.50
2018	2021	1549.33
2019	2022	1518.00

Table S2: Years with Consistently High and Low Rates of Publication Change in Metabolomics Research. Mean and standard deviation (Std) rates of change were calculated for each starting year (Year1). High-growth years (e.g., 2011–2021) were characterized by substantial adoption of metabolomics techniques, while low-growth years (e.g., 1998–2002) reflect the early development of the field. Thresholds for high and low growth were defined as the mean rate of change ± one standard deviation.

**Year1**	**Mean**	**Std**
Years with consistently high rate of change		
2011	627.57	179.10
2013	766.47	176.62
2014	796.21	214.89
2015	924.82	186.25
2016	942.88	264.33
2017	1151.25	217.46
2018	1412.38	98.406
2019	1477.12	147.45
2020	1563.06	307.18
2021	1098.75	209.66
Year1	Mean	Std
Years with consistently low rate of change		
1998	159.94	155.85
1999	176.80	161.29
2000	195.68	166.85
2001	218.96	170.63
2002	242.70	175.83

Table S3: **Publication counts by research fields in metabolomics.**
Breakdown of all publications across 18 predefined research domains, based on journal titles. The "unlabeled" category accounts for publications without domain classification.

Category	Count
unlabeled	41721
Analytical Chemistry	8981
Plant Biology	4648
Pharmacology	4229
Food Science & Nutrition	4053
Microbiology	3605
Cancer Research	2657
Environmental Science	1945
Genetics & Genomics	1312
Toxicology	1175
Neuroscience	980
Endocrinology	953
Computational Biology	943
Immunology & Vaccine Research	924
Animal Science	876
Epidemiology & Public Health	547
Aging & Gerontology	448
Developmental Biology	389
Nephrology	277
Sports Science & Medicine	76

Table S4: Publication counts by research fields in metabolomics from 1998 to 2009.

Category	Count
unlabeled	1116
Analytical Chemistry	577
Plant Biology	205
Pharmacology	155
Microbiology	109
Computational Biology	106
Genetics & Genomics	82
Food Science & Nutrition	72
Toxicology	61
Cancer Research	45
Endocrinology	21
Environmental Science	20
Neuroscience	18
Nephrology	9
Animal Science	8
Epidemiology & Public Health	7
Developmental Biology	6
Immunology & Vaccine Research	6
Aging & Gerontology	2

Table S5. **Publication counts by topics in metabolomics.**
Breakdown of all publications across the topics generated by GPT4o mini and the BERTopic pipeline. The "outlier" category accounts for publications without topic assignment.

Custom Name	Count
Outlier	14980
Plant Stress Response Mechanisms	19258
Metabolic Profiles and Dysregulation	9730
Cancer Metabolism and Therapy Resistance	7254
Metabolomics Data Analysis and Integration	6499
Gut Microbiota and Metabolomic Interactions	4313
Metabolomics in Neurodegenerative Disorders	2723
Environmental Toxicology and Metabolism	2389
Metabolomics in Animal Nutrition	1971
Microbiota-Gut-Brain Axis Interactions	1661
Kidney Disease Metabolomics and Biomarkers	1479
Metabolomics in Rheumatoid Arthritis	1318
Maternal Metabolomic Changes in Pregnancy	1238
Lung Disease and Metabolic Dynamics	1186
NMR Spectroscopy Innovations in Metabolomics	779
COVID-19 Metabolomic and Immune Responses	776
Lipid Profiling Techniques	687
Host-Parasite Metabolic Interactions	656
Male Fertility and Reproductive Metabolomics	652
Ocular Metabolomics and Disease Mechanisms	604
Salivary Metabolomics in Oral Health	503

Table S6. Per-Topic Coherence Scores for Topic Model.

GPT4o Topic Labels	C_v	C_npmi	Words
Plant Stress Response Mechanisms	0.5632	0.0360	['plant', 'plants', 'genes', 'compounds', 'stress', 'growth', 'species', 'leaves', 'biosynthesis', 'production']
Metabolic Profiles and Dysregulation	0.7128	0.1095	['liver', 'diabetes', 'insulin', 'muscle', 'mice', 'exercise', 'obesity', 'heart', 'diet', 'hepatic']
Cancer Metabolism and Therapy Resistance	0.7195	0.1224	['cancer', 'tumor', 'cells', 'cell', 'breast', 'cancer cells', 'breast cancer', 'tumors', 'mitochondrial', 'patients']
Metabolomics Data Analysis and Integration	0.5995	0.1241	['data', 'ms', 'mass', 'metabolomics', 'sample', 'method', 'biological', 'methods', 'spectrometry', 'mass spectrometry']
Gut Microbiota and Metabolomic Interactions	0.8626	0.2205	['gut', 'microbiota', 'gut microbiota', 'microbiome', 'intestinal', 'fecal', 'microbial', 'host', 'gut microbiome', 'ibd']
Metabolomics in Neurodegenerative Disorders	0.7194	0.1419	['ad', 'brain', 'alzheimer', 'alzheimer disease', 'pd', 'stroke', 'cognitive', 'csf', 'disease', 'cerebral']
Environmental Toxicology and Metabolism	0.7346	0.1281	['fish', 'zebrafish', 'exposure', 'aquatic', 'exposed', 'larvae', 'mps', 'toxicity', 'metabolism', 'effects']
Metabolomics in Animal Nutrition	0.5603	0.0767	['milk', 'meat', 'dairy', 'cattle', '05', 'feed', 'lactation', 'chicken', 'chickens', 'group']
Microbiota-Gut-Brain Axis Interactions	0.6183	0.0402	['depression', 'sleep', 'circadian', 'schizophrenia', 'gut', 'brain', 'disorder', 'antidepressant', 'microbiota', 'clock']
Kidney Disease Metabolomics and Biomarkers	0.7895	0.2373	['kidney', 'renal', 'ckd', 'kidney disease', 'chronic kidney', 'kidney injury', 'diabetic', 'injury', 'disease', 'tubular']
Metabolomics in Rheumatoid Arthritis	0.4979	0.0807	['bone', 'ra', 'mscs', 'oa', 'arthritis', 'secretome', 'osteoporosis', 'sle', 'msc', 'rheumatoid']
Maternal Metabolomic Changes in Pregnancy	0.9277	0.2883	['maternal', 'pregnancy', 'fetal', 'gestational', 'offspring', 'birth', 'women', 'pregnant', 'preterm', 'placental']
Lung Disease and Metabolic Dynamics	0.7762	0.2255	['asthma', 'lung', 'pulmonary', 'airway', 'allergic', 'respiratory', 'cf', 'chronic obstructive', 'pulmonary disease', 'obstructive pulmonary']
NMR Spectroscopy Innovations in Metabolomics	0.8594	0.4064	['nmr', 'spectra', 'spectroscopy', 'nmr spectra', 'nmr spectroscopy', 'spectral', '2d', '1d', 'magnetic', 'resonance']
COVID-19 Metabolomic and Immune Responses	0.9346	0.3981	['covid', 'covid 19', '19', 'cov', 'sars', 'sars cov', 'infection', 'viral', 'virus', 'coronavirus']

Lipid Profiling Techniques	0.6671	0.0970	['lipidomics', 'lipid', 'lipids', 'lipid species', 'lipidomic', 'ms', 'lipid classes', 'method', 'lipidome', 'mass']
Host-Parasite Metabolic Interactions	0.7282	0.1152	['parasite', 'parasites', 'infection', 'host', 'parasitic', 'infected', 'drug', 'infections', 'resistance', 'stage']
Male Fertility and Reproductive Metabolomics	0.8117	0.1191	['sperm', 'embryo', 'oocyte', 'follicular', 'embryos', 'infertility', 'seminal', 'oocytes', 'fertility', 'semen']
Ocular Metabolomics and Disease Mechanisms	0.8060	0.2262	['retinal', 'retina', 'dr', 'ocular', 'eye', 'retinopathy', 'degeneration', 'optic', 'lens', 'diabetic retinopathy']
Salivary Metabolomics in Oral Health	0.7325	0.2007	['oral', 'saliva', 'salivary', 'dental', 'saliva samples', 'salivary metabolites', 'plaque', 'biofilm', 'squamous', 'cavity']
Average	0.7311	0.1698	

Appendix A: Code Showing Label Mapping for Label Inference using Journal Titles

```python
# Define candidate labels
candidate_labels = [
    "Oncology + Cancers + Cancer + Oncogene + Anticancer + Oncotarget + Oncoimmunology + Carcinogenesis +
Metastasis + Tumori + Tumor",
    "Plant + Botany + Planta + Phytopathology + Horticulture",
    "Kidney + Nephrology + Nephron + Dialysis",
    "Endocrinology + Endocrine + Hormone + Endocrinological",
    "Microbiology + Bacteriology + Leeuwenhoek + Yeast + mBio + mSphere + Microbiome + Microbes +
MicrobiologyOpen + mSystems + Microorganisms",
    "Analytical Chemistry + Chromatography + Mass Spectrometry + Analyst + Analytica + Bioanalysis +
Separation + Spectroscopy",
    "Pharmacology + Pharmaceutical + Pharmacological + Pharmacologica + Pharmacogenomics +
Pharmacogenetics + Drug + Drugs + Ethnopharmacology + Medicinal + Natural + Pharmaceutics +
Phytopharmacology + Pharmacognosy",
    "Neuroscience + Neurochemistry + Brain + Neuroinflammation + Neurology + Neurochemical +
Neuroimmunology + Cerebral + Neuroimage + Neurotrauma + Neurological + Neuro-oncology + Neurodegeneration
+ Neuropsychiatric + Neuropsychiatry + Neuroendocrinology + Headache",
    "Nutrition + Food + Foods + Nutritional + Nutrients + Dairy + Foodborne",
    "Toxicology + Toxicological",
    "Environmental + Environment + Hazardous + Pollution",
    "Animal + Animals + Poultry + Veterinary + Livestock + Ruminant + Theriogenology + Zoology",
    "Sports + Sport + Exercise + Knee + Arthroscopy + Athletic",
    "Epidemiology + Infectious + Public",
    "Developmental + Development",
    "Gerontology + Ageing + Geriatrics + Aging + Geroscience",
    "Immunology + Immunity + Leukocyte + Autoimmunity + Immunobiology + Immunotargets + Immunotherapy +
Vaccines",
    "Bioinformatics + Chemometrics + Cheminformatics + Computational",
    "Genetics + Genomics + Genome"
]

# The label mapping
label_mapping = {
    "Oncology + Cancers + Cancer + Oncogene + Anticancer + Oncotarget + Oncoimmunology + Carcinogenesis +
Metastasis + Tumori + Tumor": "Cancer Research",
    "Plant + Botany + Planta + Phytopathology + Horticulture": "Plant Biology",
    "Kidney + Nephrology + Nephron + Dialysis": "Nephrology",
    "Endocrinology + Endocrine + Hormone + Endocrinological": "Endocrinology",
    "Microbiology + Bacteriology + Leeuwenhoek + Yeast + mBio + mSphere + Microbiome + Microbes +
MicrobiologyOpen + mSystems + Microorganisms": "Microbiology",
    "Analytical Chemistry + Chromatography + Mass Spectrometry + Analyst + Analytica + Bioanalysis +
Separation + Spectroscopy": "Analytical Chemistry",
    "Pharmacology + Pharmaceutical + Pharmacological + Pharmacologica + Pharmacogenomics +
Pharmacogenetics + Drug + Drugs + Ethnopharmacology + Medicinal + Natural + Pharmaceutics +
Phytopharmacology + Pharmacognosy": "Pharmacology",
    "Neuroscience + Neurochemistry + Brain + Neuroinflammation + Neurology + Neurochemical +
Neuroimmunology + Cerebral + Neuroimage + Neurotrauma + Neurological + Neuro-oncology + Neurodegeneration
+ Neuropsychiatric + Neuropsychiatry + Neuroendocrinology + Headache": "Neuroscience",
    "Nutrition + Food + Foods + Nutritional + Nutrients + Dairy + Foodborne": "Food Science & Nutrition",
    "Toxicology + Toxicological": "Toxicology",
    "Environmental + Environment + Hazardous + Pollution": "Environmental Science",
    "Animal + Animals + Poultry + Veterinary + Livestock + Ruminant + Theriogenology + Zoology": "Animal
Science",
    "Sports + Sport + Exercise + Knee + Arthroscopy + Athletic": "Sports Science & Medicine",
    "Epidemiology + Infectious + Public": "Epidemiology & Public Health",
    "Developmental + Development": "Developmental Biology",
    "Gerontology + Ageing + Geriatrics + Aging + Geroscience": "Aging & Gerontology",
    "Immunology + Immunity + Leukocyte + Autoimmunity + Immunobiology + Immunotargets + Immunotherapy +
Vaccines": "Immunology & Vaccine Research",
    "Bioinformatics + Chemometrics + Cheminformatics + Computational": "Computational Biology",
    "Genetics + Genomics + Genome": "Genetics & Genomics"
}
```

Appendix B: Prompt for Topic Modelling

```
# A prompt consisting of a system prompt, an in-context example, and a main prompt

openai_prompt = """
You are an expert in metabolomics and scientific literature analysis. Your task
is to generate concise, informative topic labels for collections of metabolomics
abstracts from PubMed. Each topic label should be no more than 6 words long and
should capture the essence of the metabolomics research described.

Here is an example:
I have a topic that contains the following metabolomics abstracts:
- This study investigates the metabolic profiling of plasma samples from patients
with type 2 diabetes using LC-MS/MS. We identified several key metabolites
associated with insulin resistance.
- Our research focuses on the application of NMR spectroscopy to analyze urine
samples for early detection of kidney disease. The metabolic signatures
revealed potential biomarkers.
- We employed GC-MS to examine the metabolome of cancer cells under hypoxic
conditions. The results showed significant alterations in glucose and glutamine
metabolism.

The topic is described by the following keywords: 'metabolomics, LC-MS, NMR,
biomarkers, disease detection.'

A suitable topic label would be: Disease Biomarker Discovery.

Now, based on the information provided below, please create a concise topic
label for this metabolomics topic in 6 words or fewer.

Documents: [DOCUMENTS]
Keywords: [KEYWORDS]

Return only the topic label, nothing more. Make sure it is in the following format:
topic: <topic label>
"""
```
